# Adaptive collective foraging in groups with conflicting nutritional needs

**DOI:** 10.1098/rsos.150638

**Published:** 2016-04-13

**Authors:** Alistair M. Senior, Mathieu Lihoreau, Michael A. Charleston, Jerome Buhl, David Raubenheimer, Stephen J. Simpson

**Affiliations:** 1 Charles Perkins Centre, The University of Sydney, Sydney, New South Wales 2006, Australia; 2 School of Mathematics and Statistics, The University of Sydney, Sydney, New South Wales 2006, Australia; 3 School of Life and Environmental Sciences, The University of Sydney, Sydney, New South Wales 2006, Australia; 4 Faculty of Veterinary Science, The University of Sydney, Sydney, New South Wales 2006, Australia; 5 Research Center on Animal Cognition (CRCA), Center for Integrative Biology (CBI), Toulouse University, CNRS, UPS, France; 6 School of Physical Sciences, University of Tasmania, Hobart, Tasmania 7005, Australia; 7 School of Agriculture, Food and Wine, The University of Adelaide, Adelaide, South Australia 5005, Australia

**Keywords:** foraging, individual-based model, nutritional geometry, collective decisions, social interactions, sociality

## Abstract

Collective foraging, based on positive feedback and quorum responses, is believed to improve the foraging efficiency of animals. Nutritional models suggest that social information transfer increases the ability of foragers with closely aligned nutritional needs to find nutrients and maintain a balanced diet. However, whether or not collective foraging is adaptive in a heterogeneous group composed of individuals with differing nutritional needs is virtually unexplored. Here we develop an evolutionary agent-based model using concepts of nutritional ecology to address this knowledge gap. Our aim was to evaluate how collective foraging, mediated by social retention on foods, can improve nutrient balancing in individuals with different requirements. The model suggests that in groups where inter-individual nutritional needs are unimodally distributed, high levels of collective foraging yield optimal individual fitness by reducing search times that result from moving between nutritionally imbalanced foods. However, where nutritional needs are highly bimodal (e.g. where the requirements of males and females differ) collective foraging is selected against, leading to group fission. In this case, additional mechanisms such as assortative interactions can coevolve to allow collective foraging by subgroups of individuals with aligned requirements. Our findings indicate that collective foraging is an efficient strategy for nutrient regulation in animals inhabiting complex nutritional environments and exhibiting a range of social forms.

## 1. Introduction

Many species live in heterogeneous environments in which essential resources are patchily distributed. Foraging decisions, which have a large impact on fitness, can be especially complex, leading some animals to exploit social information emanating from their conspecifics in locating and selecting foods [1,2]. In gregarious animals, such as many insects, fish, birds and ungulates, social information transfer may result in collective foraging decisions whereby all (or most) individuals in the group decide to exploit the same food resource from several available alternatives [3–5]. Typically, these collective dynamics are driven by positive feedback and quorum responses, whereby the probability of an individual choosing a resource varies positively and nonlinearly with the number of individuals already exploiting that resource [4,6]. Through these processes, groups often make faster and/or more accurate decisions than isolated animals, a phenomenon known as swarm intelligence [4,6]. To date, research on collective foraging behaviour has largely focused on identifying the mechanisms that underpin collective decision-making. However, little is known about the evolutionary roots of this widespread phenomenon [7].

Insightful data come from studies on insects, which exemplify how relatively simple social interactions, such as social retention on, or attraction to, foods based on the number of conspecifics already exploiting that food, can impact the foraging decisions and efficiency of individuals [6,8–10]. Informal comparisons of experimental data suggest that the fitness benefits of collective foraging depend on a subtle interplay between the strength of social effects in a species and the availability of resources in their environment. For instance, studies on domiciliary cockroaches (*Blattella germanica*) illustrate how the number of conspecifics already feeding on a food can influence the choice of an individual, with larger groups being more likely to attract new recruits [11,12]. It is hypothesized that this simple mechanism provides an individual cockroach with an honest signal about the quantity and/or quality of a food source enabling grouped cockroaches to make more accurate decisions than isolated conspecifics [12]. By contrast, experiments in tent caterpillars (*Malacosoma disstria*) indicate that the strength of social retention may compromise the quality of nutritional decisions, which individuals alone make with some accuracy [13]. These differential findings highlight an interesting potential trade-off between group foraging, which may increase the efficiency of individual decision-making, and the specific nutritional needs of the individual.

**Figure 1.**
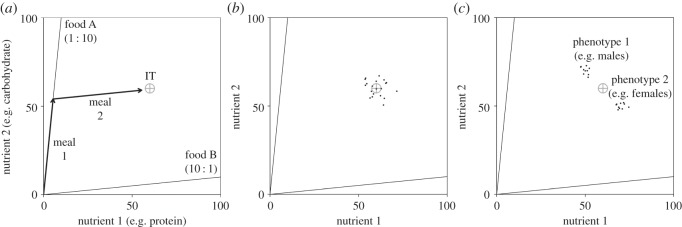
Nutritional geometry models. (*a*) Nutritional geometry (see box 1) model for two nutrients, e.g. protein on the *x*-axis and carbohydrate on the *y*-axis. In this example, the individuals' requirements are given by a single coordinate known as the intake target (IT; grey cross hair). The environment contains two foods. Food A is rich in carbohydrate (10 parts to each part protein) and food B is rich in protein. An individual has been able to move its nutritional state (NS) towards the IT, by eating food A for meal 1 and food B for meal 2 (sequence of black arrows). (*b*) While a single IT may adequately represent the mean requirements of all individuals in a group (grey cross hair), in reality there is likely to be within-group variance, with each individual having its own IT (for a hypothetical group of 20 individuals given here by black points). (*c*) In some instances, variance in nutritional requirements may be predictable. For example, if the group is composed of individuals with two phenotypes with distinct nutritional requirements (e.g. male and female), individual ITs (black points) may be bimodally distributed in two subgroups around an overall mean (grey cross hair).

Conceptual progressions in nutrition research show that foraging decisions are intrinsically complicated by the fact that individuals must take into account their needs for multiple nutrients, which may be contained in differing amounts and ratios in those foods available [14]. Ultimately studies on the evolution of collective foraging decisions must capture the complex multidimensional nature of nutrition, rather than solely focusing on the acquisition of a single resource (e.g. energy [15]). Recently, Lihoreau *et al.* [16] developed an agent-based model (ABM) derived from nutritional geometry, a state-space modelling framework for conceptualizing the nutritional decisions of animals (figure 1*a* and box 1) [14,17,18] to explore the efficiency of collective foraging in complex multi-nutrient environments. Their model suggests that an optimal level of social retention (termed *K*_soc_), whereby foragers are more likely to remain on and eat from heavily occupied food sources, improves the nutritional performance of individuals. This simple form of information transfer is sufficient to enable individuals to efficiently comprise a balanced diet from individually imbalanced, but collectively complementary, foods [18]. While this is an important first step, this approach overlooks inter-individual variation in nutritional requirements, which is likely to be present in most animal groups (cf. [15]). A recent meta-analysis exemplifies the probable ubiquity of such variability in species from different trophic levels and a wide range of taxonomic groups [24]. In part, this variation may be readily predictable based on phenotype, for example if the nutrient requirements of an individual vary with age or sex [14,25]. However, even where groups of individuals appear to be outwardly homogeneous, as for instance in a cohort of same sex individuals, heterogeneity in other traits such as metabolic rate may result in variance in nutritional requirements [24]. Given that the nutritional needs of all individuals in a group may never be perfectly aligned, does collective foraging still improve the foraging efficiency of individuals?

Box 1. **Key principles of nutritional geometry.**

Nutritional geometry is a state-based modelling approach for studying the nutritional strategies of animals, based on graphic representations of individuals, their nutritional requirements, foods and the interactions thereof in a geometric space [14,17,18]. Over recent years, this conceptual framework has become increasingly used to study how animals regulate their acquisition of multiple nutrients simultaneously, and how this varies across feeding guilds, trophic levels and taxonomic groups. In the most basic nutritional geometry models, two nutrients (such as the macronutrients protein and carbohydrate) are depicted in a two-dimensional Cartesian coordinate system forming a nutrient space (see example in figure 1*a*). An individual's nutritional state (NS) is denoted by its (*x*, *y*) coordinates, and moves through the nutrient space when foods are eaten. Foods are radials projecting through the nutrient space at angles from the origin determined by the ratio of the component nutrients contained (nutritional rails; figure 1*a*). The nutritional requirements of an individual are given by a single coordinate or a broader region within the nutrient space known as the intake target (IT, figure 1*a*) [14]. Multiple ITs may maximize different life-history traits in the same individual (e.g. maximize growth, reproduction or longevity [19,20]). However, evolutionary theory and experimental evidence suggest that animals evolve strategies that attempt to reach the IT that maximizes overall evolutionary fitness [14,19,21,22]. While most previous nutritional geometry models have assumed that a single IT would maximize the fitness of all individuals within a group or population (e.g. [23]), it is becoming increasingly clear that inter-individual variation in ITs is abundant (figure 1*b*) [24]. In the relatively homogeneous groups, this variation may be captured by a unimodal distribution of individual ITs surrounding the group mean IT (figure 1*b*). In more heterogeneous groups, these ITs may have discrete distributions, separating the group into distinct subgroups, as for example, in the case of sex-specific nutritional needs where the ITs of mixed-sex groups may be bimodally distributed (figure 1*c*).

Here we explored the costs and benefits of collective foraging for nutrient balancing in groups with increasing levels of inter-individual variance in nutritional requirements. We have developed a nutritional geometry focused ABM in which we assigned each agent an individual value of the group retention parameter *K*_soc_. Allowing *K*_soc_ to mutate across generations, we then used an evolutionary algorithm to explore how ecological and nutritional aspects of the environment affect optimal levels of group retention. Among those factors explored, we tested how aspects of inter-individual variation in nutrient needs interact with the composition and number of foods available to govern the degree of *K*_soc_ that optimizes an individual's ability to meet its nutritional requirements.

## 2. Material and methods

### 2.1. Agent-based model overview

All models were coded and simulation experiments performed in the ABM programming environment Netlogo 5.1 [26]. Model data were analysed and plotted in the statistical programming environment *R* v. 3.1.2 [27]. Below, we describe our model using the overview, design and details format as is now widely adopted for ABMs [28–30]. All Netlogo code can be found in the electronic supplementary material, S2 ‘Netlogo Code’.

#### 2.1.1. Purpose

The ABMs have been programmed to evaluate how within-group heterogeneity affects the efficacy of social retention as a mechanism to improve individual foraging in a complex nutritional environment. Previous models suggest that a relatively high level of social retention is optimal when all individuals have equal needs [15,16]. Here, we use an evolutionary algorithm to explore how this optimal level of social retention varies with inter-individual variation in nutrient requirements.

#### 2.1.2. Entities, states, variables and scales

The ABM is made up of individuals and their environment. The environment is a two-dimensional Cartesian coordinate system representing the space available for two nutrients (i.e. figure 1*a*; implemented as in Senior *et al.* [23]). Each individual's nutritional state (NS; all parameters and variables are summarized in table 1) is given by its (*x*, *y*) coordinates. An individual's fitness (*F*) is maximized when its NS reaches its intake target (IT; figure 1*a*,*b*). Individuals are given a fixed period to reach their IT before the next generation begins, and generations are non-overlapping. The group has a mean IT of (*μ_*x*_*, *μ_*y*_*) and we alter the distribution of individual ITs around this mean. An individual's NS moves as it eats. There are *N*_food_ foods in the environment, each defined by a nutritional rail: a radial projecting through the nutrient space at an angle corresponding to the nutrient balance (*V*) of that food (figure 1*a*). That is, increasing *N*_food_ increases the diversity of foods, but not their total abundance. Individuals can only eat one food at a time and thus their NS moves in parallel to the nutritional rail for the food consumed (figure 1*a*). After eating an amount of a food (*φ*), an individual may seek an alternative, based on its own nutritional requirements and the number of other individuals consuming that food. The importance ascribed to these two factors is governed by a social retention parameter, *K*_soc_, which evolves. The higher the value of *K*_soc_, the lower the probability an individual will leave a popular food. We assume that foods can be patchily distributed in space and that there is a travel time (*T*) associated with moving between foods; we have explored the effects of different values of this time-cost on the evolution of *K*_soc_. The distribution of individual ITs is governed by *σ*_IT_ and *B* (*B* is a fixed global parameter which governs the degree of bimodality in ITs; table 1, figure 1*c*). We also explored variation in ability to consume food, *φ*, although no effects were observed (see electronic supplementary material, S1 and figure S1). The model runs for 1000 generations with *K*_soc_ values randomly mutating at each generation, and *F* governing an individual's representation in the subsequent generation. Note that our aim is not to precisely mimic evolution by natural selection but to explore optimal levels of *K*_soc_ under different environments and levels of group heterogeneity.

**Table 1.** All model parameters and variables, their notation, level of operation and values (s.d. = standard deviation).

variable/parameter	notation	level	description	value
nutritional state	NS	individual	an individual's nutritional state, as tracked by its (*x*, *y*) position in the nutrient space	variable (*x*, *y*)
fitness	*F*	individual	an individual's fitness, which is a function of the Euclidean distance between an individual's NS and its IT	variable (equation (2.5))
intake target	IT	individual	an individual's IT is the (*x*, *y*) coordinate in the nutrient space that maximizes *F*	variable (*x*, *y*)
group mean IT	(*μ_*x*_*, *μ_*y*_*)	global	the mean IT (i.e. requirements) of the group for the nutrients on *x-* and *y*-axes	(500, 500)
number of foods	*N*_food_	global	the number of food types (i.e. food rails) present in environment	2, 3 or 4
nutrient balance	*V*	global	the amount of *y*-axis nutrient present within a food, relative to each part of the *x*-axis nutrient (e.g. carbohydrate to protein in figure 1*a*)	0.0625, 0.25 1, 4 and 16
capacity to eat food	*φ*	individual	the maximum amount of food that an individual can eat on one time step	2; see [25]
social retention	*K*_soc_	individual	the degree to which an individual takes into account the popularity of a food, when evaluating whether to seek an alternative	variable and evolvable
time-cost	*T*	global	the time-cost associated with finding an alternative food	0–4
standard deviation in IT	*σ*_IT_	global	the s.d. in ITs (inter-individual)	0–75
bimodality	*B*	global	the degree of bimodality in ITs (see equations (2.1) and (2.2))	0–0.5
deviation from the mean IT	ϵ	individual	the amount that an individual's IT deviates from the group mean	random-normal with mean = 0, s.d. = *σ*_IT_
distance to the IT	*D*	individual	the Euclidean distance between an individual's NS and their IT	variable
ideal food rail	*α*_ideal_	individual	the angle in radians of the ideal food rail connecting an individual's NS with their IT	variable
food ‘popularity’	*P*	individual	the proportion of the group located on the same food as a given individual	variable
assortative interactions	*A*_int_	individual	a binary variable denoting whether an individual is able to discriminate between and interact with conspecifics on the basis of their nutritional needs (1), or not (0)	0 or 1

#### 2.1.3. Process overview and scheduling

**Figure 2:**
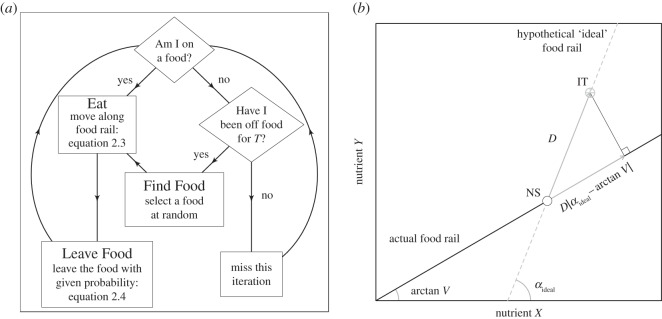
The model's implementation of nutritional geometry. (*a*) Overview of the flow of events in the agent-based model. The cycle of events is repeated 500 times, before the next generation. Generations are non-overlapping and individuals are processed randomly (see details in Material and methods). (*b*) A schematic of the model's implementation of nutritional geometry [14]. The individual's nutritional state (NS) is given as an open point, with its intake target (IT) given as a grey cross hair. The individual is eating a food with a food rail denoted by a solid black line. A hypothetical ideal food (given by the grey dashed line) would allow the individual to reach the IT. The amount of the actual food to eat (i.e. distance to move) to minimize the Euclidean distance between the individual's NS and the IT is found by multiplying *D* by the angular difference between the ideal food rail and the angle of the actual food rail (arctangent of *V*/1). All parameters and variables are given in table 1. Adapted from Senior *et al.* [23].

On each iteration of the ABM, the following three processes occur (detailed below, and in figure 2*a*): (i) ‘Find Food’, where those individuals not located on a food find one; (ii) ‘Eat’, where individuals located on a food eat some of it (i.e. move their NS through the environment); and (iii) ‘Leave Food’, where individuals may decide to leave a food on which they are feeding to seek an alternative. A new generation begins after 500 iterations. Data on the evolution of *K*_soc_ are recorded after 1000 generations.

### 2.2. Design concepts

*Basic principles*. We are interested in understanding how inter-individual variance in nutrient requirements influences optimal social retention during foraging.

*Emergence*. We are interested in the emergence of social retention, as given by the evolution of the parameter *K*_soc_. We observe how this parameter evolves under varying distributions of nutritional requirements and environments.

*Adaptation*. An individual cannot adapt, but *K*_soc_ does evolve across generations.

*Objectives*. All individuals aim to reach their IT, which is the point in nutritional space that maximizes fitness, before the end of the generation.

*Sensing*. Individuals can sense the proportion of the group that is feeding on the same food as themselves.

*Stochasticity*. Many events in the ABM occur via Bernoulli trials and certain individual traits vary by values drawn from random distributions. For example, random variation in ITs is drawn from a random-normal distribution (see below for details).

*Collectives*. Collections of individuals can form on foods, the size of which will be dependent on the current values of *K*_soc_.

*Observation*. Mean *K*_soc_ values of the entire group are observed after 1000 generations, where each generation consists of 500 model iterations.

### 2.3. Details

#### 2.3.1. Initialization

The model is initialized with 100 individuals with a NS of (0,0), and *K*_soc_ = 0. Individuals set an individual IT as given by either equation (2.1) or (2.2) with equal probability:

2.1 $$\mathrm{IT}(x,y)=(\mu_{x}+\varepsilon +\mu_{x}B,\mu_{y}+\varepsilon -\mu_{y}B)$$

and

2.2 $$\mathrm{IT}(x,y)=(\mu_{x}+\varepsilon -\mu_{x}B,\mu_{y}+\varepsilon +\mu_{y}B),$$

where *μ_*x*_* and *μ_*y*_* are the group mean requirements of the nutrients given on the *x-* and *y*-axes, *B* is a fixed global parameter taking values between 0 and 0.5 and governing the degree of bimodality in the ITs of all individuals (figure 1*c*) and *ϵ* is a value drawn from a random-normal distribution with a mean of 0 and standard deviation of *σ*_IT_. Thus, as *σ*_IT_ increases, random variation is added to an individual's IT in both the *x-* and *y*-dimensions simultaneously, and as *B* increases individual ITs separate into two subgroups.

#### 2.3.2. Find food

Those individuals that are not located on a food must find one. We assume a patchy environment, and thus every time an individual leaves a food they must spend *T* iterations searching. After *T* iterations without food an individual is randomly assigned a food from one of those available (all foods are assigned with equal probability). *T* may thus be thought of as the travel time between foods, or more conceptually how patchily foods are distributed. Given individuals have a fixed generation time to reach the IT, the inclusion of *T* thus constitutes a time-cost associated with moving between foods, which induces the selection pressure on *K*_soc_. The order of magnitude of *T* is only relevant in the context of the amount of time individuals are given to reach the IT. However, the specific value of *T* will impact the strength of selection on *K*_soc_. Previous models constrain *T* to a single value (*T* = 2 [16]), however, here we relax this constraint and explore a range of *T* values, including 0, which gives us an estimate of the evolution of *K*_soc_ in an environment where different foods are continuously distributed.

#### 2.3.3. Eat

Eating constitutes an individual moving its NS through the nutrient space in parallel to the nutritional rail of the food consumed. The distance moved (i.e. amount eaten) is given by:

2.3 $$\mathrm{distance}\;\mathrm{moved}=\min\{\varphi ,D\cos|\alpha_{\mathrm{ideal}}-\arctan V|\},$$

where *φ* is the maximum amount of food that an individual is capable of eating on one iteration, *D* is the Euclidean distance between the individual's IT and their NS, *α*_ideal_ is the angle (in radians) of a hypothetical ideal food rail connecting the individual's NS with their IT and the arctangent of *V* is the angle of the food rail of the food being consumed (a graphical representation of this model is given in figure 2*b*). Accordingly, individuals follow the ‘closest distance rule of compromise’ as defined by Simpson & Raubenheimer [14], whereby when possible individuals consume a food to minimize the Euclidean distance between their NS and IT (also see [16,23]).

#### 2.3.4. Leave food

An individual's decision to leave a food is a probabilistic function of its own nutritional requirements (i.e. whether the food will allow the individual to reach its IT), and the choice of the rest of the population. The balance between these two factors is controlled by an individual's *K*_soc_ via:

2.4 $$\mathrm{probability}\;\mathrm{of}\;\mathrm{leaving}=\max\{0.05,(1-K_{\mathrm{soc}})|\alpha_{\mathrm{ideal}}-\arctan\;V|+K_{\mathrm{soc}}e^{-7P}\},$$

where e is the base of the natural logarithm, *P* is the proportion of the group currently consuming the same food as the individual (or the food's ‘popularity’) and all other parameters are as above. Accordingly, individuals with high *K*_soc_ are highly influenced by the behaviour of the rest of the group (*P*), and individuals with lower *K*_soc_ values pay more attention to whether the food will meet their own nutritional needs (|*α*_ideal_ – arctan*V*|). We also assume that all individuals have an innate minimum probability of leaving a food of 0.05 (representing the likelihood an individual may make an imperfect decision; following [16]).

#### 2.3.5. Next generation

After 500 iterations of the above a new daughter generation begins. The fitness (*F*) of all individuals from the parental generation is calculated via

2.5 $$F=e^{-2D},$$

where all variables are as above. Thus, as individuals near their IT, their *F* approaches 1. The size of the daughter generation is fixed at 100 individuals. Each individual inherits a value of *K*_soc_ from a parent of the previous generation. At the point of inheritance (i.e. between generations), *K*_soc_ mutates by a value drawn at random from a normal distribution with a mean of 0 and standard deviation of 0.025 (and *K*_soc_ is bound at 0 and 1). All individuals in the parent group with *F* ≥ 0.25 (*F *< 0.25 is not considered fit enough to reproduce) have a probability of being selected as the parent of an individual in the daughter group proportional to *F* (for details see Senior *et al.* [23]). Individuals within the daughter generation have an initial NS of (0, 0), and define their IT following ‘Initialization’ above. The parent generation is then replaced.

## 3. Results

We varied the individual heterogeneity variables, *σ*_IT_ and *B* (values given in table 1) and measured the group mean level of *K*_soc_ at the end of 1000 generations under each set of variables. For each parameter set we performed 30 model runs and report here the mean and 0.025–0.975 quantile of the results. We explored those effects in differing nutritional environments (i.e. different *N*_food_ and *V*; table 1), and under differing assumptions about time-costs associated with finding foods (*T*).

### 3.1. Time-costs to foraging

**Figure 3:**
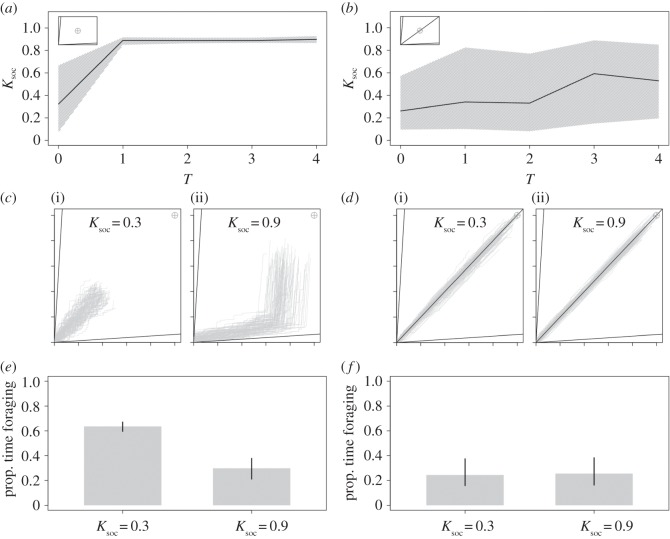
Time-costs to foraging and the evolution of collective foraging. Mean and 0.025–0.975 quantile of *K*_soc_ after 1000 generations at differing levels of *T* based on 30 model runs in (*a*) a two-food and (*b*) a three-food environment for homogeneous groups of individuals with a single IT. Embedded in each panel is a geometric depiction of the nutritional environment showing the modelled food rails (*V*). Traces of the movement of 100 agents through the nutrient space (grey lines), and their intake targets (grey cross hair) where *T* = 4 and *K*_soc_ = 0.3 (i) and *K*_soc_ = 0.9 (ii) in (*c*) a two-food environment and (*d*) a three-food environment. The mean (±0.025–0.975 quantile) proportion of that model run spent foraging (i.e. moving between foods), by those individuals in (*e*) a two-food environment and (*f*) a three-food environment. *σ*_IT_ = 0 in all instances (see table 1 for all parameters and variables).

First, we examined the efficiency of collective foraging in groups of individuals with the same IT (e.g. figure 1*a*). In an environment with two nutritionally imbalanced but complementary foods and in the absence of a time-cost to moving between foods (*T* = 0), low levels of *K*_soc_ evolved and selection was weak (i.e. highly variable values of evolved *K*_soc_; figure 3*a*). However, with any time-cost to searching for foods (*T* ≥ 1), *K*_soc_ evolved to be high and selection was strong (i.e. a narrow 0.025–0.975 quantile; figure 2*a*). At *T* ≥ 1, mean *K*_soc _= 0.89 (0.025–0.975 quantile = 0.86–0.92), which is equivalent to the optimal *K*_soc_ estimated by Lihoreau *et al.* [16] in their systematic exploration of the same parameter space. By way of contrast, with the inclusion of a third food that is nutritionally balanced relative to the group IT (rail passing through the IT), increasing *T* resulted in only slight increases in mean *K*_soc_ and in a very high variance (at *T *= 4, mean *K*_soc_ = 0.53 and 0.025–0.975 quantile = 0.20–0.85; figure 3*b*).

To explore the mechanisms underlying the evolution of social retention, we re-ran the model with fixed *K*_soc_ values (0.3 or 0.9) and *T* = 4 for a single generation. In both instances, we recorded the movements of individuals through the nutrient space and the time they spent foraging. In the two-food environment, low *K*_soc_ individuals moved frequently in order to regulate their intake of nutrients, but as a consequence spent a great deal of time foraging (figure 3*c*,*e*). By contrast, high *K*_soc_ individuals moved less frequently and took a wider path through the nutrient space (figure 3*c*). Consequently, high *K*_soc_ individuals spent roughly half as much time foraging as those with lower *K*_soc_ (figure 3*e*). In the three-food environment, all individuals spent little time foraging regardless of *K*_soc_, because they quickly found the nutritionally balanced food and no longer searched for alternatives (figure 3*d*,*f*). Ultimately, in the absence of balanced foods containing an optimal nutrient mix for all group members, high levels of social retention were selected for by reducing individual search times for nutritionally complementary resources.

Further explorations of the model suggest that increasing the number of foods (*N*_food_ = 4) selects for lower *K*_soc_. In these conditions, high *K*_soc_ forces individuals to move more often between foods, thereby precluding groups from forming on a given food, preventing foragers from efficiently tracking their IT. These effects are discussed in detail in the electronic supplementary material, S2 and figure S2.

### 3.2. Unimodal variation in individual nutrient requirements (*σ*_IT_)

**Figure 4.**
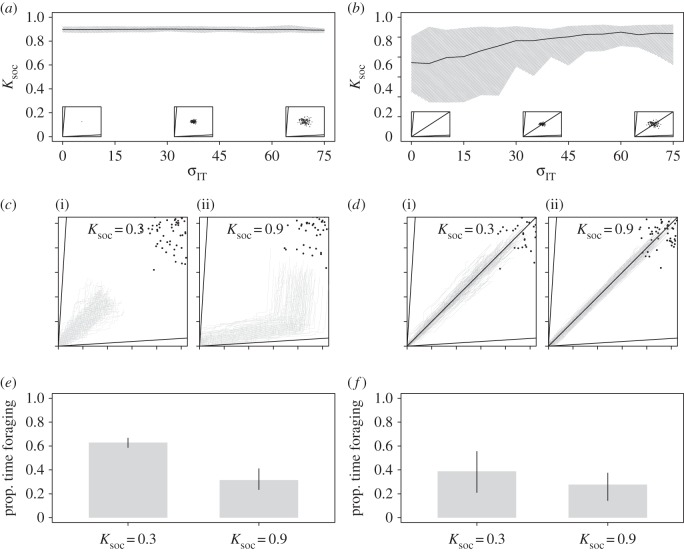
Unimodal variance in individual nutrient requirements and the evolution of collective foraging. Mean and 0.025–0.975 quantile of *K*_soc_ after 1000 generations at differing levels of *σ*_IT_ and *T* = 4 based on 30 model runs in (*a*) a two-food and (*b*) a three-food environment. Embedded in each panel is a geometric depiction of the nutritional environment showing the modelled food rails (*V*), and a visualization of variation in individual ITs at certain values (*σ*_IT_ of 0, 37.5 and 75 are shown). Traces of the movement of 100 agents through the nutrient space (grey lines) and their intake targets (black points), where *T* = 4, *σ*_IT_ = 75 and *K*_soc_ = 0.3 (i) and *K*_soc_ = 0.9 (ii) in (*c*) a two-food environment and (*d*) a three-food environment. The mean (±0.025–0.975 quantile) proportion of that model run spent foraging (i.e. moving between foods), by those individuals in (*e*) a two-food environment and (*f*) a three-food environment (see table 1 for all parameters and variables).

Next, we examined the efficacy of collective foraging in heterogeneous groups, wherein each individual had its own IT. Here, all ITs were unimodally distributed around the group mean (e.g. figure 1*b*) and there was a high time-cost to moving between foods (*T* = 4). In these conditions, no single food was balanced for all individuals. In a two-food environment, increasing variability in individual ITs (*σ*_IT_) had no effect on evolved levels of *K*_soc_ (figure 4*a*). However, the inclusion of a third food, which contained an ideal mix of nutrients for the mean requirements of the group (rail passing through the mean group IT), dramatically altered these effects. Counterintuitively, increasing *σ*_IT_ selected for increases in *K*_soc_ (figure 3*b*)*.* For example, in this three-food environment with *σ*_IT _= 0, mean *K*_soc_ = 0.54 (0.025–0.975 quantile = 0.25–0.81), but with *σ*_IT _= 75, mean *K*_soc_ = 0.84 (0.025–0.975 quantile = 0.52–0.93; figure 4*b*).

To understand the mechanisms underlying these effects, we re-ran the model and recorded the movements of individuals through the nutrient space with fixed high or low *K*_soc_ (0.3 or 0.9), *T* = 4 and *σ*_IT_ = 75 for a single generation. In the two-food environment, the benefits of group retention were high, despite a large degree of variability in individual ITs. In these conditions, high *K*_soc_ individuals spent less time foraging than low *K*_soc_ individuals (figure 4*c*,*e*). Thus, the benefits of reducing time spent foraging outweighed any benefits associated with closely tracking an individual IT. In the three-food environment, the presence of a food passing through the mean group IT (and thus being close to the requirements of most individuals) caused variability in ITs to select for group retention. Where *K*_soc_ is low, high variance in individual ITs caused individuals to leave the food that passed through the mean group IT in order to closely track their own ITs, spending a great deal of time foraging (cf. figure 3*d*,*f*, where *σ*_IT _= 0 and *K*_soc_ = 0.3, with figure 4*d*,*f*, where *σ*_IT _= 75 and *K*_soc_ = 0.3). By contrast, high *K*_soc_ (0.9) drew individuals to the food with an equal ratio of nutrients, which had a nutrient balance that was relatively close to each individual's IT (although not meeting it exactly; figure 4*d*). Being attracted to this relatively balanced food reduced the amount of time an individual spent foraging, allowing individuals to get closer to their own IT than if they moved between foods frequently (figure 4*df*). Therefore, despite high inter-individual variation in nutritional needs, social retention still enhances the ability of individuals to balance their diet from multiple complementary foods in this case.

### 3.3. Bimodality in nutrient requirements

**Figure 5.**
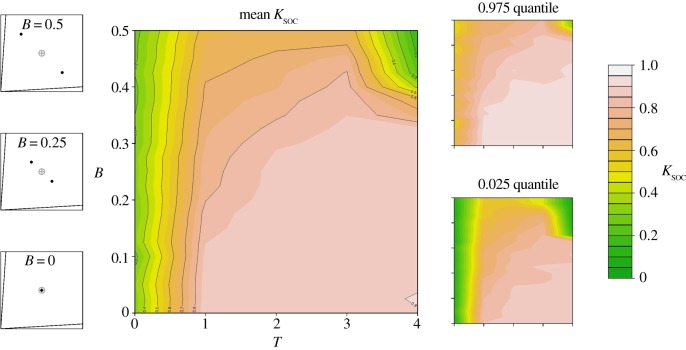
Bimodality in nutrient requirements in a two-food environment and the evolution of collective foraging. Heat maps representing the effects of covarying *B* and *T* on the mean level of *K*_soc_ after 1000 generations, in a two-food environment (0.025–0.975 quantile given on the right). Shown on the left is a nutritional geometry depiction of model settings, including the nutritional value of food rails (*V*), the mean intake target (grey cross hair) and individual intake targets (black points), distributed around the group mean with a given *B* (see table 1 for all parameters and variables).

As well as unimodal variance in nutritional requirements, we also explored a more discrete form of heterogeneity by generating groups with bimodal distributions of ITs (e.g. figure 1*c*). Overall, we detected an interaction between the degree of bimodality (*B*) in ITs and the time-cost associated with locating foods (*T*). As one would predict, in a two-food environment, increasing *B* decreased the mean level of evolved *K*_soc_. However, decreases in *K*_soc_ were only observed at higher levels of *B* (e.g. *B* > 0.25), and with low levels of *B* mean evolved *K*_soc_ remained high (figure 4). The degree to which *K*_soc_ decreased at high *B* was dependent on *T*. At low to moderate *T* (i.e. *T* = 1–3), high *B* only slightly reduced the evolved level of *K*_soc_ (mean *K*_soc_ ≈ 0.7; figure 5). However, where both *T* and *B* were high, evolved *K*_soc_ was low (e.g. *T* = 4 and *B* = 0.5, mean *K*_soc_ ≈ 0.3; figure 5).

The presence of a third food rail that meets the mean group IT (but of none of the individual ITs) produced different results (shown in the electronic supplementary material, figure S3). In general, in highly bimodal groups, collective foraging is not an efficient strategy, especially in environments containing foods with balances of nutrients that meet the mean requirements of the group.

### 3.4. Assortative interactions

A simple mechanism to overcome the constraints that discrete distributions in ITs place on the evolution of social retention may be *assortative interactions*, whereby group members only forage with those that share their nutritional needs. To explore this, we included a trait that allows individuals to discriminate between conspecifics on the basis of their ITs (*A*_int_; table 1), which may coevolve with *K*_soc_. We assume that *A*_int_ is a binary and heritable trait, where *A*_int_* *= 1 allows individuals to interact only with those that share their needs, and *A*_int_ = 0 does not (the probability of mutating from one state to the other was 0.01). Given that there may be time-costs associated with the processing of information for evaluating individuals prior to making a foraging decision, we also assume that individuals with *A*_int_ = 1 take an additional iteration to locate a food source (i.e. the time-cost of moving between foods = *T* + *A*_int_).

**Figure 6.**
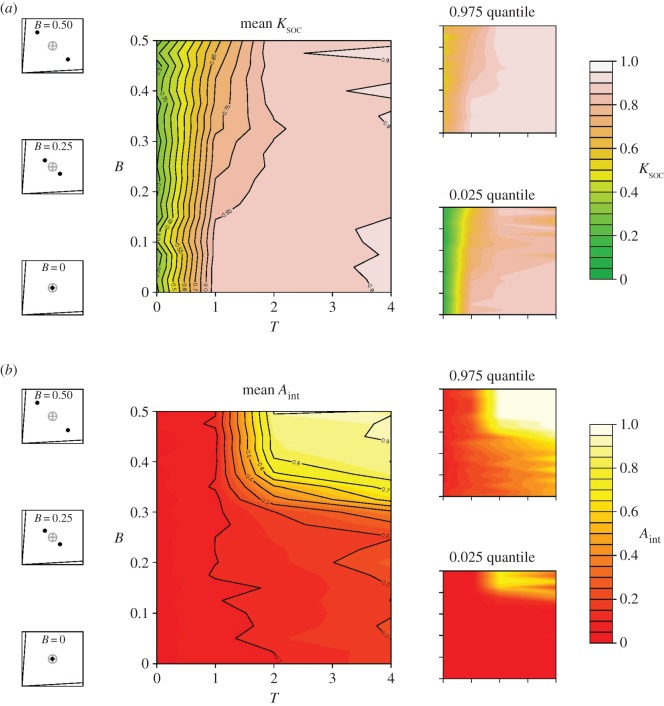
Bimodality in nutritional requirements and the co-evolution of collective foraging and assortative interactions. Heat maps represent the effects of covarying *B* and *T* on the mean (0.025–0.975 quantile given on the right) level of (*a*) *K*_soc_ and (*b*) *A*_int_ after 1000 generations, when the two traits are allowed to co-evolve in a two-food environment (i.e. equivalent of figure 4). Shown on the left is a nutritional geometry depiction of model settings, including the nutritional value of food rails (*V*), the mean intake target (grey cross hair) and individual intake targets (black points), distributed around the group mean with a given *B* (see table 1 for all parameters and variables).

We found that where *A*_int_ could evolve, the constraints that *B* had on the evolution of *K*_soc_ were overcome. For example, in a two-food environment with high *T* and high *B* (4 and 0.5, respectively), and where *A*_int_ and *K*_soc_ can coevolve, *K*_soc_ evolved to around 0.9 (figure 6*a*). This result contrasts with the evolved *K*_soc_ of 0.2 obtained under equivalent model setting where *A*_int_ could not evolve (figure 4). We also found selection for *A*_int_, despite the associated cost. Where *B* and *T* are high, *A*_int_ evolved to be around 0.9 on average, but was selected against in all other areas of the parameter space (figure 6*b*). Thus, time-costs associated with foraging as well as distinct bimodality in nutrient requirements may select for the evolution of assortative foraging, whereby groups segregate into subgroups of individuals with similar nutritional needs. See the electronic supplementary material, S3, figures S4 and S5, for equivalent results in three- and four-food environments.

## 4. Discussion

Many animals, from insects to mammals, make collective decisions that enhance the speed and/or accuracy of individual choices [5,31,32]. While previous studies show how collective foraging enables individuals to select the richest or the largest available food resources in their environment [8–10], little is known about whether and how individuals can also collectively regulate their acquisition of vital nutrients from multiple imbalanced foods [15,16]. Our evolutionary model, based on concepts of nutritional geometry, indicates that collective foraging is often an efficient strategy for nutrient balancing, even in groups of individuals with different needs.

### 4.1. Collective foraging is adaptive in heterogeneous groups

In cases where nutrient intake must be balanced from complementary foods and there is a time-cost to foraging, our model shows that social retention is an effective mechanism for preventing individuals from over-investing in the time spent foraging. This prediction remained true in a number of instances where the specific requirements of individuals actually varied, only appearing to be suboptimal when the group contained an incredibly high degree of bimodality (i.e. figure 5). However, the presence of a food that met the mean requirements of the group (i.e. a food rail passing through the mean group IT) interacted with the distribution of ITs to determine optimal levels of social retention. When such a food was present and intra-group variation in ITs was low, social retention was only weakly selected for, despite costs associated with foraging. Surprisingly, in this environment, increasing inter-individual variance around a single mean (and therefore increasing potential conflict of interest between individuals) selected for stronger social retention (i.e. figure 4*b*). Here, collective foraging caused individuals to converge on a food that would meet their needs approximately, rather than over-investing (in terms of foraging time) in attempting to accurately cater to the idiosyncrasies of their own dietary requirements. Ecological scenarios such as those identified by our model may help explain how group retention can evolve to be so strong that it effectively traps individuals on nutritionally imbalanced foods, overriding their own nutritional needs, such as has been observed in the forest-tent caterpillar [13], where experimental conditions do not necessarily match natural nutritional environments.

Given the above, we can make a number of predictions about where organisms might, or might not, be expected to display collective foraging based on simple mechanisms of group retention. Namely, our model states that social retention becomes maladaptive under three circumstances: (i) where there is no cost associated with nutrient balancing (e.g. foods are nutritionally balanced or not patchily distributed); (ii) when the environment becomes increasingly complex (large numbers of different types of foods patchily distributed), and individuals are too sparsely scattered across patches to effectively aggregate; and (iii) when the group has a highly bimodal distribution of requirements. Accordingly, we may expect organisms that inhabit stable environments falling under one, or more, of the above categories not to display social retention, whereas in the reciprocal environment the trait should evolve. Testing such a prediction would necessitate cross-taxa comparative study. However, in organisms that live in fluctuating environments, we might expect individuals to update and adapt their use of social information in response to environmental change [2,33]. For such species, nutritional geometry provides an experimental framework with which to evaluate the use of social information for nutrient balancing. Foods with known nutritional composition can be created, allowing one to manipulate the composition and distribution of foods in the environment [14]. In addition, if the species does not intrinsically contain individuals with differing nutritional needs it may be possible to experimentally modify the distribution of inter-individual requirements by manipulating individual NS (e.g. feeding individuals with differing foods prior to the trial).

### 4.2. Assortative foraging and group heterogeneity

In the extreme case where inter-individual variance has a highly discrete (multimodal) distribution, for instance, when groups are composed of distinct subsets of individuals with divergent needs, social retention tended to be selected against. Bimodal distributions of ITs may be common in mixed groups composed of males and females. Such observations have been made, for example, in field crickets (*Teleogryllus commodus*) [25] and fruit flies (*Drosophila melanogaster*) [34,35], where females maximize their reproductive fitness on diets richer in protein than males. Our results suggest that where the differences between sexes in nutritional requirements are large, collective foraging may be selected against. However, the constraints that sex-specificity in nutritional requirements place on the evolution of group foraging are easily overcome if individuals interact in an assortative manner, for instance by preferentially following individuals of the same sex rather than the group as a whole.

Sexual segregation of foraging groups has been observed in taxa ranging from marine and terrestrial mammals to fish [36–38]. Numerous competing hypotheses have been proposed to explain this behaviour [38], and comparative analyses restricted to large herbivores have provided perhaps the best insights. A leading hypothesis, termed the ‘forage selection’, or ‘nutritional needs’ hypothesis, explicitly states that sexual segregation results from differential dietary requirements of the sexes [37]. While support from comparative studies for this hypothesis has been mixed [37,39], data have focused on looking for differences in ‘diet-quality’ (e.g. nitrogen content) between males and females, rather than differences in diet composition *per se* (i.e. required ratios and amounts of nutrients, or ITs). Our model suggests that bimodality in nutritional requirements, in combination with selective pressures that favour collective foraging (e.g. time-costs associated with foraging), can lead to the emergence of such sexual segregation.

### 4.3. Collective nutrient regulation and the evolution of group complexity

It has long been suggested that nutritional constraints, such as limited access to key nutrients, may promote the evolution of cooperation and division of labour in animal groups [40–42]. However, the lack of a conceptual framework for testing these hypotheses has long hampered such research. Our theoretical exploration of the costs and benefits of social retention across multiple levels of group heterogeneities suggests that nutritional factors such as spatial/temporal distribution of foods and individual nutrient requirements are potential drivers of the evolution of collective foraging. Recently, it has been proposed that levels of intra-group variance in ITs and NSs may become greater with increasing degrees of social complexities, as one moves from forming temporary aggregations to living in permanent and fully eusocial colonies that forage from a central sedentary location [15]. Group living may increase inter-individual variance in requirements because of the simultaneous effects of competition over nutrient acquisition, age structures or differential parental nourishment, all of which are associated with cooperation and division of labour [43–45]. Ultimately, these factors may lead to the evolution of highly integrated social groups containing multiple classes of individuals with discrete ITs, as for instance the different castes of individuals that characterize the colonies of eusocial insects (e.g. ants, bees, wasps, termites) [46]. Although not its primary aim, our model predicts an evolutionary relationship between group heterogeneity (as measured by inter-individual variance in ITs) and social interactions (collective foraging) that is modulated by the nutritional environment (nutrient balance of available foods), partially supporting the above hypothesis.

Our results further suggest that collective foraging is not selected for in groups composed of individuals with highly divergent nutritional needs (i.e. bimodal distribution of ITs). However, this does not necessarily mean that intra-group variance is incompatible with sociality. As we have shown, in such species, additional mechanisms can evolve to mitigate the costs of collective foraging. For instance, eusocial insects have evolved sophisticated collective regulatory behaviour whereby a subset of individuals (the foragers) attempts to collect amounts and balances of nutrients that address the divergent nutritional needs of all colony members. In these advanced societies, foragers are able to gather foods that meet the high protein requirements of larvae and the queens, as well as the other adults that are more reliant on carbohydrates, based on a complex system of nutritional feedback between colony members [47–51].

### 4.4. Further development and conclusions

Our model, derived from nutritional geometry, provides a theoretical platform for exploring the costs and benefits of collective foraging in complex nutritional environments. A major advantage of this approach is that it generates specific predictions that can be empirically tested using well-established experimental designs from nutritional geometry [18]. Using this framework, it is possible to combine behavioural observations of groups of interacting animals feeding on chemically defined diets and to correlate individual nutrient intakes with measures of fitness traits both at the individual and collective levels [18]. Our model assumes a perhaps simplistic nutritional environment (i.e. foods are not finite and do not shift spatially), as our focus is primarily on heterogeneity in individual needs, in an environment that can be experimentally replicated. Future developments of our multidimensional approach, however, could integrate spatio-temporal distributions of finite foods constituting different compositions of essential nutrients, which ultimately determine the probability of a forager locating resources [52,53]. These factors constitute perhaps the most realistic costs to foraging in complex and ecologically relevant environments. Temporal and spatial variability in food abundance will alter the value of social information, thus affecting the nutritional strategy or strategies that will be optimal, both for an individual and groups. What is more, finite food sources will intrinsically capture aspects of intra-group conflict over a limiting resource, which will further affect the cost–benefit trade-off of group living.

Previous models demonstrate that simple mechanisms of social retention can optimize nutrient balancing in groups with homogeneous nutritional requirements. Here we present the first evolutionary ABM that re-evaluates the efficacy of such mechanisms for groups with heterogeneous nutritional needs. Our model suggests that simple mechanisms of social retention can improve foraging efficiency by reducing foraging times, even in relatively heterogeneous groups. Further, counterintuitively in some nutritional environments, heterogeneity in nutritional needs may select for increased collective decision-making. However, in groups with highly bimodal distributions of nutritional requirements (e.g. where needs of the sexes differ greatly) additional mechanisms of assortative foraging must coevolve alongside mechanisms of group retention, a potential route to the evolution of sexual segregation. Finally, our model may imply an evolutionary relationship between nutritional requirements, the ecological costs associated with foraging and the evolution of sociality, and provides a powerful framework for investigating such relationships.

## Supplementary Material

File S1: Supplementary Results

## Supplementary Material

File S2: Netlogo Code
