# Supplementary material for: Adaptive collective foraging in groups with conflicting nutritional needs
Source: R Soc Open Sci. 2016 Apr 13;3(4):150638. doi: 10.1098/rsos.150638 (PMC4852629; doi:10.1098/rsos.150638)
Supplement: File S1: Supplementary Results [file rsos150638supp1.doc]

## Supplementary Results

### Text S1. Variance in Ability to Consume Food

In addition to inter-individual variance in ITs, one may also envisage a scenario in which there is inter-individual variance in the amount of food an individual can eat in a given time period. Here the maximum amount an individual can eat per iteration is given by *φ* (see Table 1 for a list of all parameters and variables). We explored a model where the amount of food each agent can eat is governed by an individual level parameter with the value drawn from a random-normal distribution with a mean of *φ* and standard deviation of *σ_φ_* at the beginning of each generation. We considered homogeneous groups where all individuals had the same IT. In a two-food environment containing two individually imbalanced but collectively complementary foods relative to the group IT, increasing *σ_φ_* had no effect on mean *K*_soc_ (Fig. S1.A). In a three-food environment, which contained an additional optimally balanced food relative to the group IT, *σ_φ_* had no effect on the evolution of mean *K*_soc_ at *T* = 0, although variance in *K*_soc_ decreased slightly at higher values of *σ_φ_* (Fig. S1.B). At *T* = 4, increasing *σ_φ_* caused a decrease in mean *K*_soc_, although the effect was slight and variance in evolved *K*_soc_ remained high (Fig. S1.B).

### Fig. S1

Mean and 0.025 to 0.975 quantile of *K*_soc_ after 1000 generations at differing standard deviation in the amount individuals can eat on any one iteration (*σ_φ_*), based on 30 model replicates in homogenous groups where all individuals have the same IT for A) a two-food environment and B) a three-food environment. Embedded in the top left of each figure is a geometric depiction of the nutritional environment showing the modeled food rails (*V*). Black *T* = 0 and red *T* = 4 and *σ*_IT_ = 0 in all instances (see Table 1 for all parameters and variables).

### Text S2. Time Costs to Foraging in Four-Food Environments

We explored the efficacy of social retention (*K*_soc_) as a mechanism to increase individual foraging efficiency in a four-food environment, which contained two pairs of complementary foods relative to the single group IT (*V* = 0.0625, 0.25, 4 and 16). In this environment increasing the time costs (*T*) associated with moving between foods increased the evolved level of *K*_soc_ (Fig. S2.A). However, at values of *T* > 1, social retention was selected against and very low values of mean *K*_soc_ with a low variance evolved (Fig. S2.A). To explore why *K*_soc_ was selected against at high values of *T*, we re-ran the model for one generation with all individuals expressing either low *K*_soc_ (0.3) or high *K*_soc_ (0.9). We recorded the progression of each individual’s nutritional state through the nutrient space and time spent foraging with *T* = 4. High *K*_soc_ individuals had a slightly slower progression through the nutrient space than low *K*_soc_ individuals, as they spent more time foraging (Figs S2.B and S2.C). A high level of social retention forced individuals to move more often than if they had low social retention. This is presumably because there were too many available resources for any single food to build up a majority of foragers at a given time. Rather, individuals ended up thinly distributed across several foods, causing high *K*_soc_ individuals to constantly search for a large group with which to aggregate.

### Fig. S2

(A) Mean and 0.025 to 0.975 quantile of *K*_soc_ after 1000 generations at differing levels of *T* in a four-food environment where all individuals have the same IT based on 30 model replicates (*σ*_IT_ = 0 in all instances). Embedded is a geometric depiction of the nutritional environment showing the modelled food rails (*V*). (B) Traces of the movement of 100 agents through the nutrient space (grey lines), and their intake target (grey cross hair) where *T* = 4 and *K*_soc_ = 0.3 (left) and *K*_soc_ = 0.9 (right). (C) The mean (± 0.025 to 0.975 quantile) proportion of that model run spent foraging (i.e. moving between foods; see Table 1 for all parameters and variables).

###
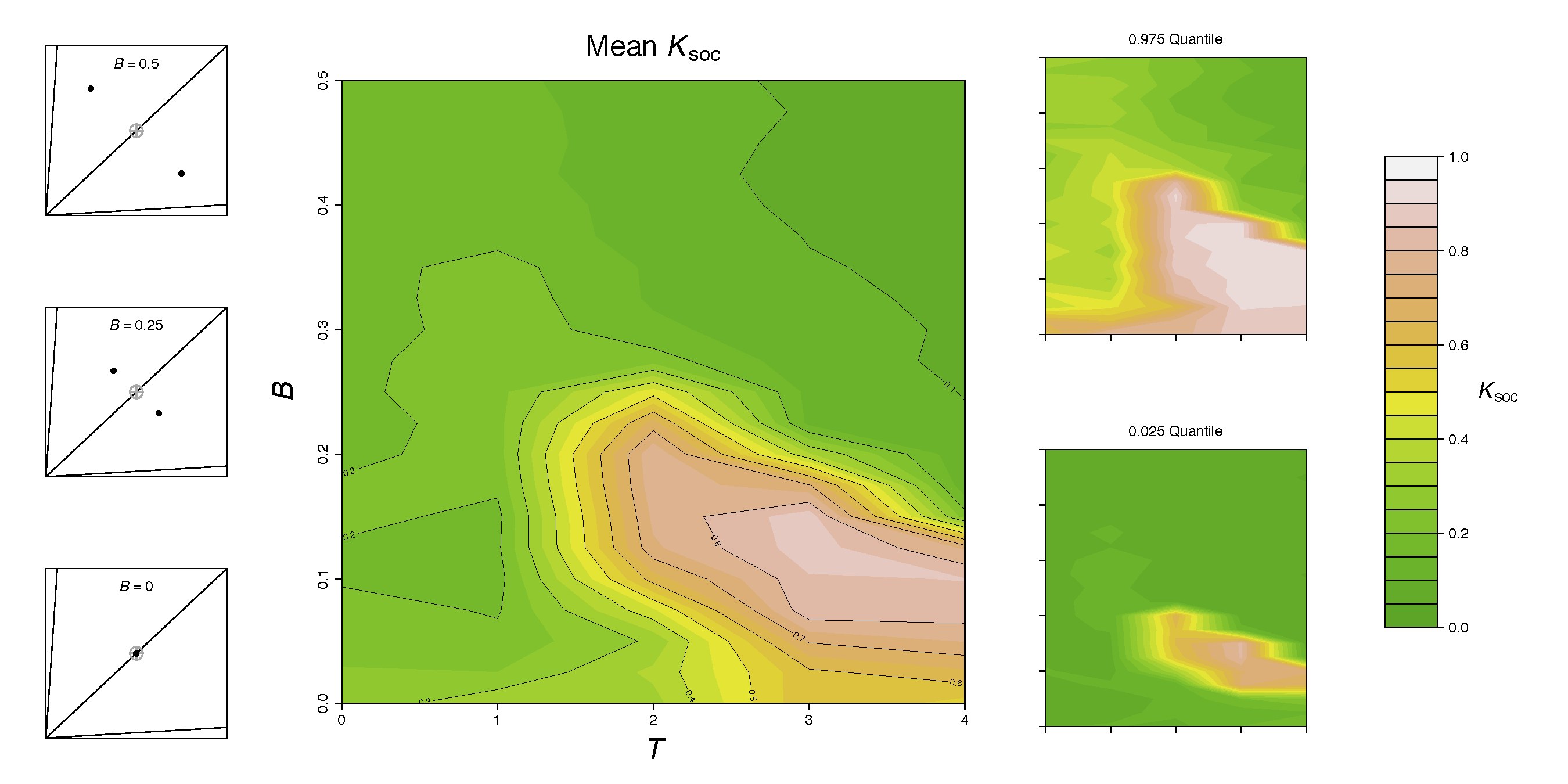
Fig. S3

Heat maps representing the effects of co-varying *B* and *T* on the mean level of *K*_soc_ after 1000 generations, in a three-food environment (0.025 to 0.975 quantile given on the right). Shown on the left is a nutritional geometry depiction of model settings, including the nutritional value of food rails (*V*), the mean intake target (grey cross hair) and individual intake targets (black points), distributed around the group mean with a given *B* (see Table 1 for all parameters and variables).

### Text S3. Assortative Interactions (*A*_int_) in Three-Food and Four-Food Environments

We explored whether *K*_soc_ (see Table 1 for all parameters and variables) and *A*_int_, coevolve in groups where ITs are bimodally distributed in three- and four-food environments. Models were implemented as previously (see Methods and Results in the Main Text). We considered heterogeneous groups with bimodally distributed individual ITs. In the three-food environment, which contained a food with a nutritional rail that met the mean requirements of the whole group (that is, it passed through the mean group IT), we found that the ability of individuals to evolve conspecific discrimination, mediating associative foraging did not alter the effect of *B* or *T* on *K*_soc_ (compare the evolution of *K*_soc_ in Fig. S3 with Fig. 5). In addition we did not observe any mean *A*_int_ value higher than 0.5, in the three-food environment (Fig. S3). A similar trend was observed in the four-food environment, which contained two pairs of nutritionally imbalanced but complementary foods relative to the mean group IT. Here, *K*_soc_ only evolved to high values in a very small portion of the parameter space, and *A*_int_ did not appear to evolve at all (Fig. S4). In these increasingly complex nutritional environments social retention is only an effective mechanism for increasing nutrient regulation efficiency under very specific circumstances, regardless of whether individuals can evolve conspecific discrimination.

###
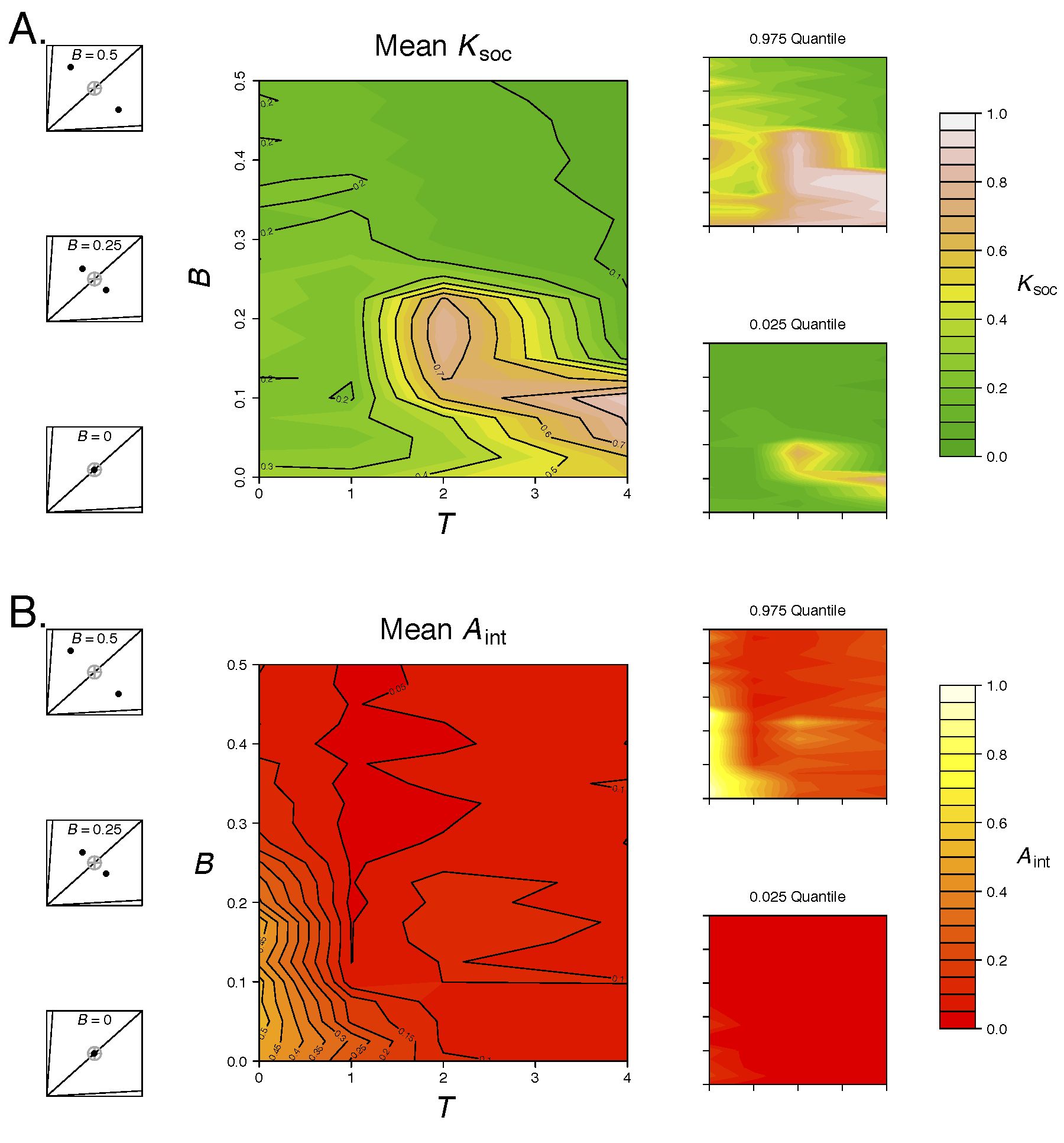
Fig. S4

Heat maps represent the effects of co-varying *B* and *T* (see Table 1 for all parameters and variables) on the mean level of (A) *K*_soc_ and (B) *A*_int_ after 1000 generations, when the two traits are allowed to co-evolve in a three-food environment (0.025 to 0.975 quantile given on the right). Shown on the left is a nutritional geometry depiction of model settings, including the nutritional value of food rails (*V*), the mean intake target (red cross hair) and individual intake targets (black points), distributed around the group mean with a given *B.*

###
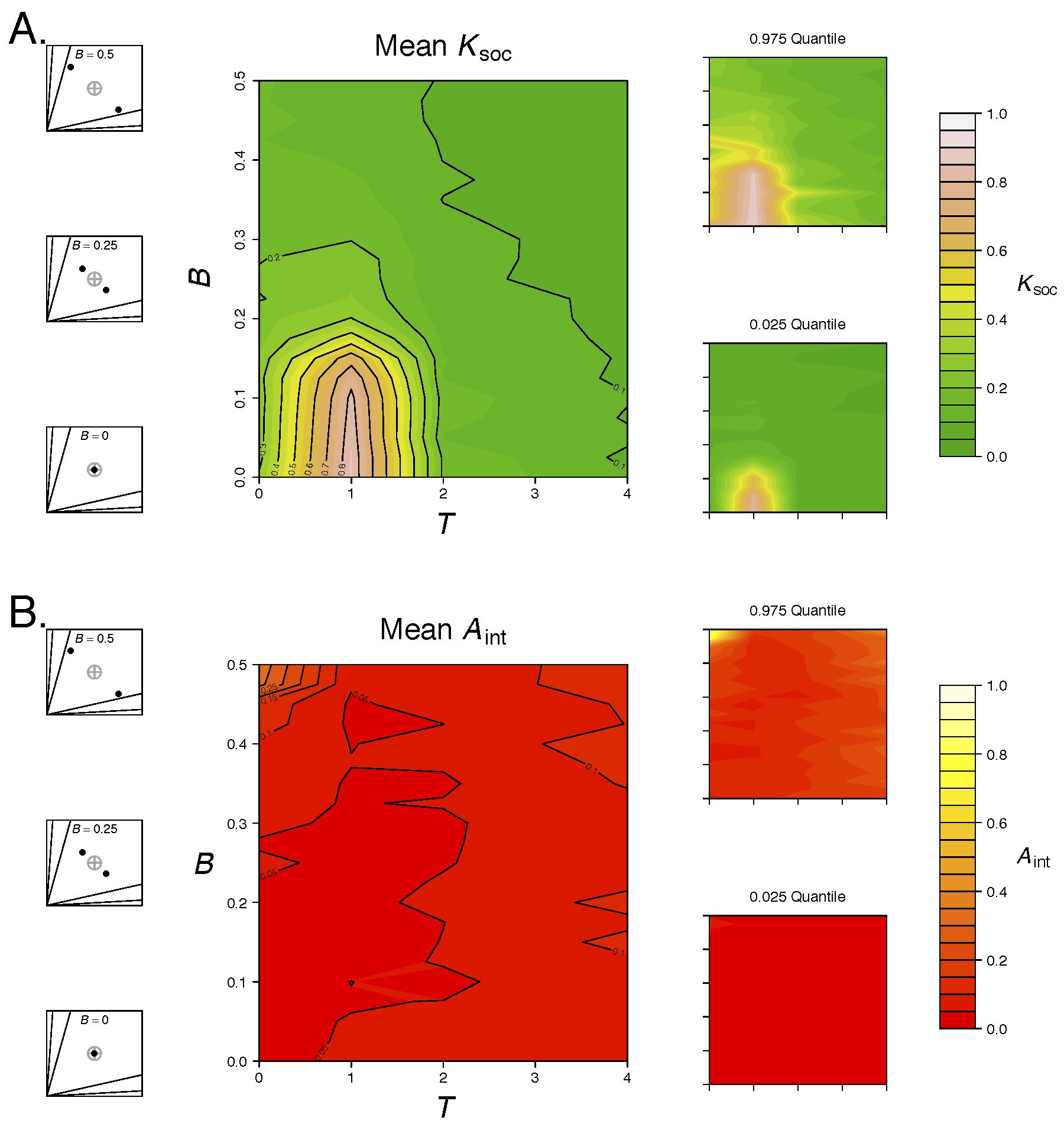
Fig. S5

Heat maps represent the effects of co-varying *B* and *T* (see Table 1 for all parameters and variables) on the mean level of (A) *K*_soc_ and (B) *A*_int_ after 1000 generations, when the two traits are allowed to co-evolve in a four-food environment (0.025 to 0.975 quantile given on the right). Shown on the left is a nutritional geometry depiction of model settings, including the nutritional value of food rails (*V*), the mean intake target (red cross hair) and individual intake targets (black points), distributed around the group mean with a given *B.*
