# Supplementary material for: Adaptive collective foraging in groups with conflicting nutritional needs
Source: R Soc Open Sci. 2016 Apr 13;3(4):150638. doi: 10.1098/rsos.150638 (PMC4852629; doi:10.1098/rsos.150638)
Supplement: File S2: Netlogo Code [file rsos150638supp2.rtf]

;;; This is Netlogo 5.1 code written to accompany Senior et al. 2015. Collective Decision Making Enhances Nutrient Regulation in Complex Environments and Heterogeneous Groups;;; The code was written by A. M. Senior @ the University of Sydney in 2015;;; Detailed information about the biological justification for the model can be found in the accompanying MS;;; Other similar models can be found in Lihoreau et al. 2014, 2015 and Senior et al. 2015 - see MS for the full citations;;; To run this code;;; 1) Download Netlogo (https://ccl.northwestern.edu/netlogo/);;; 2) Copy and paste all of this code under the 'code' tab;;; 3) YOU MUST also on the interface tab create the following;;; * two sliders called 'FOOD.A' and 'FOOD.B', with range 0 - 16;;; * two switches called 'record' and 'Evolve-Aint';;; * a slider called 'T', with range 0 - 4;;; * a slider called 'ITSD', with range 0 - 75;;; * a slider called 'B' with range 0 - 0.5;;; * a slider called 'phiSD' with range 0 - 0.5;;; * the usual netlogo buttons, 'setup', 'go' (forever) and 'go';;;;;;;;;;;;;;;;;;;;;;;;;;;;;;;;;;;;;;;;;;;;;;;;;;;; PARAMETERS ;;;;;;;;;;;;;;;;;;;;;;;;;;;;;;;;;;;;;;;;;;;;;;;;;;;;;;;;;;;;;;;;;;;;;;;;; Below are the global level parameters/variables. ;;; The species has an average required protein and carbohydrate intake target; ProteinIT and CarbIT. These are values specified as coordinates in [x,y] space.;;; Protein is the position on the X-axis and Carbs is on the Y. Also not mentioned, but represented by sliders are the variables FOOD.A and FOOD.B, which determine, the slope of carb on ;;; protein for each food in the environment: i.e. for one unit protein intake, how much carb does a food contain. ;;; The varibles FOOD.X-heading tell a turtle how to navigate through the nutrient space on the basis of one of the foods it is eating. ;;; alpha-FOOD.X is the angular distance between the food being consumed and the x-axis (as described in Lihoreau et al 2014 and Senior et al. 2015);;; 'dead' records whether the population has gone extinct (i.e. no individuals reached a high enough fitness to breed);;; 'generations' records how many the generations the model has been running for;;; 'parents' is a list of all the possible parents in the population, from which individuals in the next generation select a mother;;; evolved-Ksoc and evolved-Aint record the mean Ksoc and Aint of all individuals in the population;;; pinnate  globals [      ProteinIT  CarbIT    FOOD.A-heading  alpha-FOOD.A  FOOD.B-heading  alpha-FOOD.B    dead  generations  parents    evolved-Ksoc  evolved-Aint  ];;; Patches have no specified parameters, but the inbuilt location of a patch - x and y is very important in this modelpatches-own [];;; Below are the turtles individual level paramters / variables;;; 'appetite', 'alpha-f', 'alpha-ideal', 'beta' and 'Ndist' are used in the models implementation of nutritional geometry.;;; Appetite determines how much of a given FOOD a turtle would need to eat to maximise it's fitness (F) on that food. ;;; the variables alpha-f and alpha-ideal as are described in Lihoreau et al. 2014 and Senior et al. 2015 - which in turn are used to calulate beta ;;; NOTE that depending on the food being consumed ('choice'), alpha-f will be alpha-FOOD.X;;; N-Dist and beta are the used to calculate appetite. Appetite is the scaler projection of the vector between the individual and there intake target (alpha-ideal) on to the food rail;;; full determines whether a turtle has been able to eat it's appetite. Only those turtles that are not full (i.e. full = 0) will keep eating;;; F is fitness and is calulated as the inverse (kind-of) of the euclidean distance (Edist) between the turtles current location and the intake target. As the turtle gets closer to IT the higher it's fitness gets;;; pInd and pSoc, determine the probability that an individual leaves a food (pLeave), based on their own nutirtional needs and the behaivour of the group.;;; time-out is a counter that determines how long an individual has spent 'moving between foods';;; Individuals then have their own IT, which deviates from that of the group mean by a random amount, and is called my.ProteinIT and my.Carb.IT, based on ITSD and B;;; ITs seperate in to two broups based on B and IT.Type records which groups the individual falls in to (needed for Aint);;; phi governs the maximum amount of food an individual can eat - in most analyses there is no variation between individuals in this parameter;;; Ksoc (continuous between 0 and 1) is the individual-level of group retention - how an individual trades-off pInd and pSoc;;; Aint (discrete value of 0 OR 1) is the individual level of assoratative interaction;;; Eval is an individuals evolutionary value - F normalized over the entire population;;; 'mother' is an individuals parent;;; ALSO NOTE: colours are used to distinguish between turtles of different types. orange turtles are those that are located on a food and still will eat it. Pink turtles are;;; those that are located on a food, but do not want to keep eating. blue turtles are those that are not located on a food, i.e. moving between foods.turtles-own [        appetite  alpha-f  alpha-ideal  beta  Ndist  choice  full    F  Edist    pInd  pSoc  pLeave    time-out    my.ProteinIT  my.CarbIT  IT.Type    phi    Ksoc  Aint    Eval  mother  ] ;;;;;;;;;;;;;;;;;;;;;;;;;;;;;;;;;;;;;;;;;;;;;;;;;;::;;; SET-UP ;;;;;;;;;;;;;;;;;;;;;;;;;;;;;;;;;;;;;;;;;;;;;;;;;;;;;;;;;;;;;;;;;;;;;;;;to setup     ;;; Create a world    clear-all    resize-world 0 1000 0 1000  set-patch-size 0.75    ;;; Create the group mean intake target of protein and Carb and make it a red cross  ;;; For simplicity I assume a balance between protein and carb    set ProteinIT 500   set CarbIT 500  ask patches with [pxcor = ProteinIT and pycor > (CarbIT - 10) and pycor < (CarbIT + 10)] [set pcolor red]   ask patches with [pycor = CarbIT and pxcor > (ProteinIT - 10) and pxcor < (ProteinIT + 10)] [set pcolor red]    ;;; there have been no generations yet, and the population is not extinct  set generations 0  set dead 0    ;;;;;;;;;;;;;;;;;;;;;;;;;;;;;;;;;;;;;;;;;;;     ; Visualise the rail for FOOD.A (based on the slider FOOD.A) as green lines by asking patches   ; associated with the correct protein:carb ratio 'turn green'. If the world is resized (e.g. if the IT is moved)   ; the values of 700 below will need to be adjusted.    if(FOOD.A <= 1)[    foreach n-values 1000 [?]    [      ask patch ? (? * FOOD.A) [set pcolor green]     ]  ]    if(FOOD.A > 1)[    foreach n-values 1000 [?]    [      ask patch (? * 1 / FOOD.A) ? [set pcolor green]     ]  ]        ; Set the details about FOOD.A i.e. it's heading in the netlogo world and it's alpha as described in Lihoreau et al and the MS.     if(FOOD.A <= 1)[set FOOD.A-heading [towards patch 1000 (1000 * FOOD.A)] of patch 0 0]  if(FOOD.A > 1)[set FOOD.A-heading [towards patch (1000 / FOOD.A) 1000] of patch 0 0]    set alpha-FOOD.A subtract-headings 90 FOOD.A-heading      ; Same for Food.B    if(FOOD.B <= 1)[    foreach n-values 1000 [?]    [      ask patch ? (? * FOOD.B) [set pcolor green]     ]  ]    if(FOOD.B > 1)[    foreach n-values 1000 [?]    [      ask patch (? * 1 / FOOD.B) ? [set pcolor green]     ]  ]      if(FOOD.B <= 1)[set FOOD.B-heading [towards patch 1000 (1000 * FOOD.B)] of patch 0 0]  if(FOOD.B > 1)[set FOOD.B-heading [towards patch (1000 / FOOD.B) 1000] of patch 0 0]    set alpha-FOOD.B subtract-headings 90 FOOD.B-heading      ; NOTE REPLICATE the above for an ith food.    ;;;;;;;;;;;;;;;;;;;;;;;;;;;;;;;;;;;;;;;;;;;     ;;; Create 100 turtles  ;;; each set their IT   ;;; Each sets a food a food at random and records info about that food  ;;; set all of your othe rindividual level paramters accordingly    crt 100 [        set xcor 0    set ycor 0    set size 10        set time-out 0        ifelse(random-float 1 > 0.5)    [          set my.ProteinIT ProteinIT + (CarbIT * B) + random-normal 0 ITSD      set my.CarbIT CarbIT - (ProteinIT * B) + random-normal 0 ITSD      set IT.Type "A"    ]    [      set my.ProteinIT ProteinIT - (CarbIT * B) + random-normal 0 ITSD      set my.CarbIT CarbIT + (ProteinIT * B) + random-normal 0 ITSD      set IT.Type "B"    ]         set choice one-of ["A" "B"]        if (choice = "A")      [        set heading FOOD.A-heading        set alpha-f alpha-FOOD.A        set color orange      ]    if (choice = "B")      [        set heading FOOD.B-heading         set alpha-f alpha-FOOD.B        set color orange      ]        set alpha-ideal subtract-headings 90 towards patch my.ProteinIT my.CarbIT    set Edist (sqrt (((xcor - my.ProteinIT) ^ 2) + ((ycor - my.CarbIT) ^ 2))) / (sqrt (((0 - my.ProteinIT) ^ 2) + ((0 - my.CarbIT) ^ 2)))        set F 0        set Ndist sqrt(((my.ProteinIT - xcor) ^ 2) + ((my.CarbIT - ycor) ^ 2))        set time-out 0    set color orange        set Eval 0    set mother nobody        set phi (sqrt 2) * ((sqrt (((0 - ProteinIT) ^ 2) + ((0 - CarbIT) ^ 2))) / 500) + random-normal 0 phiSD        set Ksoc 0        ask patch my.ProteinIT my.CarbIT  [set pcolor white]        set full 0    set Aint 0  ]          ; if we are recording data from the model at the end of each generation, create that file.    if (record)[    if (file-exists? "data.csv")    [carefully [file-delete "data.csv"]      [print error-message]]        file-open "data.csv"        file-type "Generation,"    file-type "Mean Ksoc of Individuals,"    file-type "Standard Deviation of Ksoc,"    file-type "mean Aint of Individuals,"    file-type "Standard Deviation of Aint,"    file-type "Mean Fitness of Indviduals,"    file-print "SD of Fitness"    file-close      ]      ; reset the ticks    reset-ticks  end;;;;;;;;;;;;;;;;;;;;;;;;;;;;;;;;;;;;;;;;;;;;;;;;;;;;;; GO ;;;;;;;;;;;;;;;;;;;;;;;;;;;;;;;;;;;;;;;;;;;;;;;;;;;;;;;;;;;;;;;;;;;;;;;;;;;;;; Turtles not located on a food (blue), find a food.;;; The turtles that are not full and are on a food (orange) eat and re-calculate their fitness;;; turtles on food, may choose to leave and find an alternative;;; After 500 cycles of this, there is a new generation;;; If the population goes extinct or there have been 1000 generations, stop the simulationto go    ask turtles with [color = blue and full = 0] [FIND_FOOD]  ask turtles with [color = orange and full = 0] [EAT]  ask turtles with [color = orange or color = pink and full = 0] [LEAVE_FOOD]    tick    if (ticks = 500) [    NEXT_GENERATION  ]       if (dead = 1) [stop]  if (generations = 1000) [stop]    end;;;;;;;;;;;;;;;;; find-food ;;;;;;;;;;;;;;;;;;;;;;;;;;;;;;;;;;;;; turtles must spend a given amount of time (time-out) moving between or 'looking for' foods.;;; once they have spent that much time out, they select one of the foods at random, and record info about that foodto FIND_FOOD    ifelse (time-out > 0)    [      set time-out time-out - 1    ]    [      set choice one-of ["A" "B"]            if (choice = "A")      [        set heading FOOD.A-heading        set alpha-f alpha-FOOD.A        set color orange      ]      if (choice = "B")      [        set heading FOOD.B-heading         set alpha-f alpha-FOOD.B        set color orange      ]    ]  end;;;;;;;;;;;;;;;;;;;;;;;;;;;;;;;;;;;;;;;;;; EAT ;;;;;;;;;;;;;;;;;;;;;;to EAT    ;;; Calculate intakes in netlogo world angles (based on 0degrees north),   ;;; then convert in to the angle alpha-ideal as described the MS (0 degrees is X-axis) - also see Lihoreau et. al 2014 and Senior et. al 2015    set alpha-ideal subtract-headings 90 towards patch my.ProteinIT my.CarbIT    ;;; You then calulate your appetite; the scalar projection of the IT from your current location   ;;; on to the food rail you're on by taking beta - the angluar difference between alpha-f and alpha-ideal.    set beta abs subtract-headings alpha-f alpha-ideal  set Ndist sqrt(((my.ProteinIT - xcor) ^ 2) + ((my.CarbIT - ycor) ^ 2))    set appetite Ndist * cos beta    ;;; Either eat your appetite, or eat phi. If you have just eaten your appetite,   ;;; you will likely need to see if an alternative food is 'any better' i.e. can it get you closer to the IT  ;;; Note you cannot move backwards so if your appetite suggests you should, look for a different food.    ifelse (appetite >= phi) [    fd phi  ]  [    ifelse(appetite < 0)      [        set color pink      ]      [        fd appetite        set color pink      ]      ]    if (xcor = my.ProteinIT and ycor = my.CarbIT) [set full 1]    end;;;;;;;;;;;;;;;;;;;;;;;;;;;;;;;; LEAVE_FOOD ;;;;;;;;;;;;;;;;;;;to LEAVE_FOOD    ;;; The probability of leaving a food is dependant of pLeave (as described in the paper), which is a composite of your individual needs (pInd) and   ;;; the social effect (pSoc), moderated by Ksoc, the paramter of interest.  ;;; There is also an innate probaility of leaving of 0.05. The actual probability evaluated is what ever is the maximum of the innate and calculated values     set pInd (abs ((alpha-ideal * (pi / 180)) - (alpha-f * (pi / 180)))) / (pi / 2)    ;;; Note that if you have Aint (1), you base pSoc on only those individuals that share your IT type, but if you leave your time-out will be higher    if (Aint = 0) [set pSoc exp (-7 * count turtles with [alpha-f = [alpha-f] of myself] / count turtles)]  if (Aint = 1) [set pSoc exp (-7 * count turtles with [alpha-f = [alpha-f] of myself and IT.Type = [IT.Type] of myself] / count turtles with [IT.Type = [IT.Type] of myself])]    set pLeave ((1 - Ksoc) * pInd) + Ksoc * pSoc    ifelse (pLeave > 0.05)  [    if(random-float 1 < pLeave)    [      set time-out T + Aint      set alpha-f 0      set color blue    ]   ]  [    if(random-float 1 < 0.05)    [      set time-out T + Aint      set alpha-f 0      set color blue    ]      ]    end;;;;;;;;;;;;;;;;;;;;; NEXT-GENERATION -- Restart a new generation; the evolutionary algorithm in action ;;;;;;;;;;;;;;;;;;;;;;;;;;;;;;;;;;;;;;;;to NEXT_GENERATION    ;;; Calculate the evolved values of the two traits    set evolved-Ksoc mean [Ksoc] of turtles  set evolved-Aint mean [Aint] of turtles     ;;; Reset the color of your personal IT, so we can see next generations ITs  ask turtles [ask patch my.ProteinIT my.CarbIT  [set pcolor black]]    ;;; calculate fitness (F). Note that in Lihoreau et al. 2014 the distance between initialisation and the IT is always 1   ;;; So here, for the fitness function to be equivalent, we must rescale the netlogo distances from the current nutritional state to the IT   ;;; by the total distance between the IT and the point of initialisation    ask turtles [set Edist (sqrt (((xcor - my.ProteinIT) ^ 2) + ((ycor - my.CarbIT) ^ 2))) / (sqrt (((0 - my.ProteinIT) ^ 2) + ((0 - my.CarbIT) ^ 2)))]  ask turtles [set F exp (-2 * Edist)]    ;;; if we are recording data then do that  if (record) [record-data]    ;;; get rid of any individuals with a low fitness    ask turtles with [F < 0.25] [die]    ;;; if there is less than 2 individuals left record the simulaiton as an extinction event and stop    if (count turtles < 2)[    set dead 1    stop  ]    ;;; calculate the normalised fitness values for individuals    ask turtles [set Eval F / sum [F] of turtles]    ;;; create the list of available parents.  set parents sort turtles    ;;; create the next generation; i.e. the offspring and give them the parameters in the [ ].    crt 100 [        set xcor 0    set ycor 0    set size 10        set time-out 0        ifelse(random-float 1 > 0.5)    [          set my.ProteinIT ProteinIT + (CarbIT * B) + random-normal 0 ITSD      set my.CarbIT CarbIT - (ProteinIT * B) + random-normal 0 ITSD      set IT.Type "A"    ]    [      set my.ProteinIT ProteinIT - (CarbIT * B) + random-normal 0 ITSD      set my.CarbIT CarbIT + (ProteinIT * B) + random-normal 0 ITSD      set IT.Type "B"    ]        set alpha-f 0    set alpha-ideal subtract-headings 90 towards patch my.ProteinIT my.CarbIT        set Edist (sqrt (((xcor - my.ProteinIT) ^ 2) + ((ycor - my.CarbIT) ^ 2))) / (sqrt (((0 - my.ProteinIT) ^ 2) + ((0 - my.CarbIT) ^ 2)))        set F 0        set choice one-of ["A" "B"]        if (choice = "A")      [        set heading FOOD.A-heading        set alpha-f alpha-FOOD.A        set color orange      ]    if (choice = "B")      [        set heading FOOD.B-heading         set alpha-f alpha-FOOD.B        set color orange      ]        set Ndist sqrt(((my.ProteinIT - xcor) ^ 2) + ((my.CarbIT - ycor) ^ 2))        set time-out 0    set color orange        ;;; set parent using fitness-proportionate selection, on the basis of the of normalised fitness values         set mother random-weighted parents map [[Eval] of ?] parents         ;;; set you own Ksoc, that of the selected parent, with mutation.        set Ksoc ([Ksoc] of mother + random-normal 0 0.025)        ;;; bound K at 0 and 1        if (Ksoc > 1) [set Ksoc 1]    if (Ksoc < 0) [set Ksoc 0]        set phi (sqrt 2) * ((sqrt (((0 - ProteinIT) ^ 2) + ((0 - CarbIT) ^ 2))) / 500) + random-normal 0 phiSD        ;;; get a Aint value from your parent, and if we are evolving that, mutate it        set Aint [Aint] of mother        if (Evolve-Aint)[            if (random-float 1 < 0.01)[        ifelse (Aint = 0)        [set Aint 1]        [set Aint 0]      ]        ]        set Eval 0    set mother nobody        ask patch my.ProteinIT my.CarbIT  [set pcolor white]        set full 0  ]        ; Kill of the last generation  ask turtles with [xcor > 0] [die]  reset-ticks    ; Reset the patch colors for the population level IT  ask patches with [pxcor = ProteinIT and pycor > (CarbIT - 10) and pycor < (CarbIT + 10)] [set pcolor red]   ask patches with [pycor = CarbIT and pxcor > (ProteinIT - 10) and pxcor < (ProteinIT + 10)] [set pcolor red]    ; count the number of generations iterated over  set generations generations + 1    end; the program to record data at the end of a generation if we are doing this.to record-data    file-open "data.csv"    file-type (word generations ",")  file-type (word mean [Ksoc] of turtles ",")  file-type (word sqrt (variance [Ksoc] of turtles) ",")  file-type (word mean [Aint] of turtles ",")  file-type (word sqrt (variance [Aint] of turtles) ",")  file-type (word mean [F] of turtles ",")  file-print (word sqrt (variance [F] of turtles))    file-close  end;;;;;;;;;;;;;;;;;;;;;;;;;; Random-weighted selector for mother and father selection; This is the random weighted selector used for fitness-proportionate selection.; Netlogo does not have a random-weighted selector built in. Thus I always use the following.; a reporter to return a random value from a list based upon a weight assigned; the reporter works based on cumulative inverse probability and takes two lists; the first a list to choose from,; the second is a list of corresponding weights for each of those entries in the first list; it sets selector to be a random number based on the sum of the weights and sets a running sum to be zero.; then for each weight in the list, it cumulatively adds them until they exceed the selector; the first value in the first list to have it's weight exceed selector is returned; this reporter was taken from net-logo users group : user = Nick Bennett; http://groups.yahoo.com/group/netlogo-users/message/9091to-report random-weighted [values weights]   let selector (random-float sum weights)   let running-sum 0       (foreach values weights [       set running-sum (running-sum + ?2)       if (running-sum > selector) [         report ?1       ]     ]) end 
